# Gene expression in peripheral mononuclear cells from septic patients secondary to community-acquired pneumonia: patterns of gene expression and outcomes

**DOI:** 10.1186/cc12665

**Published:** 2013-06-19

**Authors:** P Severino, E Silva, GL Baggio-Zappia, MKC Brunialti, O Rigato, IDC Guerreiro da Silva, FR Machado, R Salomao

**Affiliations:** 1 Hospital Israelita Albert Einstein, Morumbi, São Paulo, SP, Brazil

## Introduction

Sepsis is defined as a systemic inflammatory response secondary to a proven or suspected infection. Mechanisms governing this inflammatory response have been shown to be complex and dynamic, involving cross-talk among diverse signaling pathways. However, current knowledge on mechanisms underlying sepsis is far from providing a complete picture of the syndrome, justifying additional efforts that might add to this scenario. Microarray-based expression profiling is a powerful approach for the investigation of complex clinical conditions such as sepsis: the analysis of gene transcription at the genome level potentially avoids results derived from biased assumptions. In this study we investigate whole-genome gene expression profiles of mononuclear cells from survivor and nonsurvivor septic patients.

## Methods

Blood samples were collected at the time of sepsis diagnosis and 7 days later, allowing us to evaluate the role of biological processes or genes possibly involved in patient recovery. Aiming to circumvent, at least partially, the heterogeneity of septic patients, we included only patients admitted with sepsis caused by community-acquired pneumonia. Global gene expression profiling allowed us to characterize early sepsis, as compared with healthy individuals.

## Results

Our results corroborate literature reports on inflammation response in the early stages of sepsis but highlight great heterogeneity in gene expression during the onset of sepsis. Differences in oxidative stress seem to be associated with clinical outcome, since significant differences in the expression profile of related genes were observed between survivors and nonsurvivors. However, our results substantiate current knowledge supporting that sepsis syndrome development is indeed multifaceted. Although the initial infection of enrolled patients was pneumonia, with no sign of organ failure at the time of diagnosis, 7 days later gene expression profiles seemed to be characteristic for each patient, with no clear pattern of development. This result could be associated with the underlying health status of each one of them, with complications due to sepsis itself as well as with distinct timing for response to treatment.

## Conclusion

At this point we conclude that studies should focus on studying sepsis at multiple time points, aiming to capture common, although possibly separated in time when patients are considered, biological processes associated with recovery.

